# 外周T细胞淋巴瘤非特指型合并纯红细胞再生障碍1例报告并文献复习

**DOI:** 10.3760/cma.j.cn121090-20240809-00297

**Published:** 2025-04

**Authors:** 学亚 张, 瑜瑜 郑, 大铬 范, 金发 钟, 诗馨 吴

**Affiliations:** 1 福建医科大学附属第二医院血液科，泉州 362000 Department of Hematology, the Second Affiliated Hospital of Fujian Medical University, Quanzhou 362000, China; 2 福建医科大学附属第二医院检验科，泉州 362000 Department of Laboratory, the Second Affiliated Hospital of Fujian Medical University, Quanzhou 362000, China; 3 福建医科大学附属第二医院病理科，泉州 362000 Department of Pathology, the Second Affiliated Hospital of Fujian Medical University, Quanzhou 362000, China

## Abstract

外周T细胞淋巴瘤非特指型（PTCL-NOS）合并纯红细胞再生障碍（PRCA）国内报道较少。本文报道1例PTCL-NOS患者，以重度贫血为首发表现，伴有多部位淋巴结肿大。实验室检查证实红细胞减少、网织红细胞比例降低、骨髓红细胞增生明显减低，骨髓CD3^+^CD8^+^ T淋巴细胞比例显著增高，淋巴结病理及免疫组化符合PTCL-NOS，TCRβ基因重排阳性，一线ECHOP方案化疗4个疗程后，患者肿大淋巴结显著缩小，全身CT评估部分缓解，血红蛋白升至正常，骨髓造血红系增生恢复正常，CD3^+^CD8^+^ T淋巴细胞未检出，目前口服来那度胺维持治疗，预后尚好。

## Abstract (en)

Nonspecific peripheral T-cell lymphoma（PTCL-NOS）combined with pure red cell aplasia（PRCA）is reported by only few in China. This article described a patient with PTCL-NOS who presented with severe anemia as the initial symptom, accompanied by multiple lymphadenopathy. Laboratory tests confirmed decreased red blood cell count and reticulocyte proportion, markedly reduced bone marrow red blood cell proliferation, and markedly increased proportion of bone marrow CD3^+^CD8^+^ T lymphocytes. On lymph node pathology examination, immunohistochemistry was consistent with PTCL-NOS, with positive TCRβ gene rearrangement. First-line chemotherapy with four courses of ECHOP regimen led to significant reductions in lymph node size, partial remission on whole-body computed tomography evaluation, normalization of hemoglobin and bone marrow hematopoietic erythroid proliferation, and absence of CD3^+^CD8^+^ T lymphocytes in bone marrow. Currently, the patient remains to have good prognosis with maintenance oral lenalidomide.

外周血T细胞淋巴瘤（PTCL）属于恶性血液系统肿瘤，占非霍奇金淋巴瘤的10％～20％，包括20多种临床病理亚型，呈侵袭性且预后不良，只有半数患者经过规范治疗获得长期生存[1]。非特指型是外周T细胞淋巴瘤最常见的组织学亚型，中老年多见，多发生在淋巴结，绝大多数患者同时表现为结外受累，包括肝、骨髓、皮肤和胃肠道[1]。

纯红细胞再生障碍（pure red cell aplasia，PRCA）是一种临床较为少见的贫血性疾病，其特征是伴严重网织红细胞减少的正细胞正色素贫血，骨髓中幼红细胞显著减少或缺如，WBC和PLT正常[2]–[4]。该病常见于成人，儿童人群少见，可分为先天性和继发性。继发性PRCA常与自身免疫炎症、感染性疾病或肿瘤疾病相关，如胸腺瘤、T细胞大颗粒淋巴细胞白血病（T-LGLL）、人类细小病毒B19感染、EB病毒（EBV）感染等[5]–[7]。本院收治1例外周T细胞淋巴瘤非特指型（PTCL-NOS）合并PRCA患者，其经过ECHOP方案化疗，取得了良好的效果。

## 病例资料

患者，男，59岁。因“面色苍白伴乏力、活动后气促1个月”于2024年1月就诊我院。患者入院前1个月无明显诱因出现面色苍白、乏力，伴活动后气促，当地医院血常规：WBC 7.24×10^9^/L、HGB 48 g/L、PLT 376×10^9^/L；全身CT扫描：纵隔、双肺门及双侧腋窝、腹膜后、盆腔及双侧腹股沟多发肿大淋巴结。予输注悬浮红细胞支持治疗后，转诊至我院进一步诊治。入院查体：神志清楚，重度贫血外观，颈部双侧、腋下、腹股沟均可触及淋巴结肿大，质地中等，活动性一般，无压痛。胸骨无压痛，双肺呼吸音清，未闻及干湿性啰音，心律齐，各瓣膜听诊区未闻及杂音，腹平坦，无压痛、反跳痛，肝脾肋缘下未触及，双下肢无水肿。入院后血常规：WBC 5.74×l0^9^/L、RBC 1.56×10^12^/L、HGB 46 g/L、平均红细胞体积86.4 fl、平均红细胞血红蛋白量29.7 pg、平均红细胞血红蛋白浓度341 g/L、PLT 361×10^9^/L、网织红细胞比例0.1％，外周血涂片未见破碎红细胞。叶酸、维生素B_12_浓度、血清铁蛋白均正常；男性肿瘤标志物、肝肾功能、CD55、CD59均未见异常；传染病四项：阴性；甲状腺功能三项正常；抗核抗体、ENA谱无异常；人类细小病毒B19阴性；Coombs试验：（+++）；EBV-DNA及巨细胞病毒DNA阴性；骨髓涂片：增生活跃，粒红比为96∶1，红系增生极度低下，占0.5％，巨核细胞22个；淋巴细胞比例增高，占49％，胞质易见数量不等的粗大紫红色颗粒；单核细胞比例正常，形态未见明显异常。诊断意见：骨髓象示粒系比例正常、红系偶见、淋巴细胞增多。骨髓流式细胞术免疫分型，在CD45/SSC点图上设门分析，淋巴细胞约占有核细胞的57％，其中CD3^+^CD8^+^ T淋巴细胞约占淋巴细胞的79.5％，比例明显增高，CD4^+^/CD8^+^细胞比值明显降低；原始细胞约占有核细胞的0.5％，散在分布；单核细胞约占有核细胞的2.5％，表型成熟粒细胞约占有核细胞的39％，比例降低，未见明显发育异常。分子生物学检测显示，T细胞受体（TCR）β基因重排阳性，未检出免疫球蛋白（Ig）基因重排。染色体核型分析显示46，XY[20]。右髂后上棘骨髓病理：增生活跃（70％～80％），粒红比例增大，粒系各阶段细胞可见，以中幼及以下阶段细胞为主；红系各阶段细胞可见，以中晚幼红细胞为主；巨核细胞正常，分叶核为主；淋巴细胞及浆细胞增多，散在簇状分布，网状纤维染色（MF-1级）；免疫组化结果：病灶内细胞CD3（较多+）、CD5（较多+）、CD20（散在+）、PAX5（散在+）、CD138（较多+）、CD23（−）、CD10（−）、Cyclin D1（−）、TdT（个别+）、CD117（少数+）、MPO（粒系+）。右侧腋下淋巴结病理：淋巴结基本结构破坏，可见少量残存的淋巴滤泡，并可见较多血管增生；高倍视野下异常淋巴细胞增生，胞体小，胞质少，核圆形或不规则，染色质粗，其间可见较多浆细胞浸润；免疫组化结果：CD3（+）、Ki-67（20％～30％+）、CD4（+）、CD8（−）、CD20（−）、CD21（残留FDC+）、CD138（浆细胞+）、Kappa（较少+）、Lambda（较多+）、ICOS（散在+）、PD1（散在+）、CD30（散在+）、EBER（散在+）。诊断：结合形态学及免疫组化结果考虑PTCL-NOS。

PET-CT检查：颈部双侧包括枕部、耳前、耳后、腮腺旁，见多发肿大淋巴结，部分融合成团，最大者位于右侧V区，约2.4 cm×1.5 cm，伴^18^F-氟代脱氧葡萄糖（FDG）摄取异常增高，最大标准摄取值（SUVmax）为4.1；双侧腋窝及双侧肩区见多发肿大淋巴结，部分融合成团，最大者位于左侧腋窝，约2.4 cm×1.6 cm，伴^18^F-FDG摄取异常增高，SUVmax为4.6；纵隔（2R、3A、4R、4L、5、6、7区）及双肺门见多发肿大淋巴结，部分融合成团，最大者位于4R区，约2.8 cm×1.8 cm，伴^18^F-FDG摄取异常增高，SUVmax为4.8；肝门区、双侧膈肌脚内侧、肠系膜、腹膜后见多发肿大淋巴结，部分融合成团，最大者位于腹主动脉左侧旁，约2.7 cm×1.5 cm，伴^18^F-FDG摄取异常增高，SUVmax为5.8；盆腔两侧见多发肿大淋巴结，部分融合成团，最大者位于左侧，约3.2 cm×1.8 cm，伴^18^F-FDG摄取异常增高，SUVmax为5.5；双侧腹股沟区见多发肿大淋巴结，最大者位于右侧，约2.2 cm×1.5 cm，伴^18^F-FDG摄取异常增高，SUVmax为4.3；双侧腘窝见多发肿大淋巴结，最大者位于右侧，约1.5 cm×0.8 cm，伴^18^F-FDG摄取异常增高，SUVmax为4.5。结论：全身多发肿大淋巴结，伴^18^F-FDG代谢异常增高，结合病史，符合淋巴瘤浸润。全身骨骼未见明显骨质破坏，关节结构正常。

综合以上检查结果，明确诊断患者为PTCL-NOS合并PRCA，单纯予以输注悬浮红细胞支持治疗，并于2024年3月开始行ECHOP方案（依托泊苷100 mg·m^−2^·d^−1^，第1～3天；环磷酰胺750 mg/m^2^，第1天；表阿霉素70 mg/m^2^，第1天；长春新碱2 mg，第1天；地塞米松15 mg/d，第1～5天；1个疗程）化疗4个疗程，化疗过程顺利。其中1个疗程化疗结束后患者出现肛周疼痛、发热，予以抗感染、升白细胞处理，患者肛周疼痛缓解，白细胞恢复正常。患者1个疗程后乏力症状改善，2个疗程后，HGB明显上升至96 g/L，复查Coombs试验阴性；4个疗程后HGB升至116 g/L，骨髓红系比例由治疗前0.5％上升至20％；骨髓流式细胞术检测未见异常CD3^+^CD8^+^ T淋巴细胞。心、肺、肝、肾功能正常。全身CT评估疗效为部分缓解。目前患者口服来那度胺维持治疗，HGB处于正常范围，多普勒超声及CT检查显示淋巴结大小较前无变化，提示病情控制良好。

## 讨论及文献复习

该患者有贫血症状和体征，无出血、发热和肝脾肿大，血常规提示重度贫血，网织红细胞比例小于1％，WBC和PLT正常，骨髓造血提示红系造血细胞比例为0.5％，根据《血液病诊断及疗效标准（第4版）》[4]，患者明确诊断为PRCA。PRCA是一种红系孤立缺失的自身免疫性疾病，通常分为先天性和获得性两大类，获得性又可分为继发性和原发性两种。根据患者既往病史，先天性可排除[8]–[11]。获得性查不到具体病因，则考虑原发性；而继发性可见于多种疾病[3]。根据PubMed检索显示较多与血管免疫母细胞性T细胞淋巴瘤相关的PRCA病例报告[12]–[14]，这些报告中几乎所有患者的直接Coombs试验呈阳性，这表明自身反应抗体在PRCA发病中的作用；在这种情况下，由于严重贫血和直接Coombs试验阳性、补体C3、C4活性减低，应考虑同时存在自身免疫性溶血性贫血（AIHA）。但根据临床发现，我们不认为患者患有AIHA：首先，没有溶血的临床证据，如黄疸、脾肿大、间接胆红素、LDH升高；其次，外周血涂片检查结果与AIHA不一致，没有破碎红细胞或红细胞碎片的证据；再次，患者的其他临床表现与AIHA不一致，如该患者显示出极低的网织红细胞比例且在其他正常骨髓中几乎完全没有成红细胞，Ig基因重排阴性，同时骨髓流式细胞术免疫分型未见B淋巴细胞增多。

患者染色体核型检测正常，可排除以PRCA为早期表现的骨髓增生异常综合征；另外，T-LGLL是一种罕见的细胞毒性淋巴细胞克隆性增殖性疾病，大颗粒淋巴细胞因含有数量不等的嗜苯胺蓝颗粒而得名，典型表型为CD3^+^CD8^+^CD57^+^CD4^-^CD56^-^CD28^-^ TCRαβ^+^［4]，该病也可合并PRCA、TCR基因重排阳性，但本例患者骨髓形态学特点及临床特征和细胞免疫表型不支持T-LGLL诊断。考虑到患者多发淋巴结肿大，及时行淋巴结活检术，根据术后淋巴结病理免疫组化结果以及TCR重排阳性，明确诊断患者为PTCL-NOS，经过一线ECHOP方案化疗后，患者全身肿大淋巴结明显缩小，HGB正常，骨髓红系比例显著升高至正常值，取得了良好的疗效，符合PTCL-NOS相关的PRCA诊断，这与国内其他中心报道的PTCL-NOS合并PRCA病例的诊断类似，但缺乏CD3^+^CD8^+^ T淋巴细胞的数据[15]–[17]。另外，不同于已报道的病例接受ECHOP和MINE联合泼尼松方案以及一线ECHOP方案联合泼尼松治疗[15]–[17]，本例通过单纯ECHOP方案化疗，取得了PRCA的完全缓解，同时伴有CD3^+^CD8^+^ T淋巴细胞比例的动态改变和TCR基因重排转阴，提示动态检测PTCL-NOS合并PRCA患者CD3^+^CD8^+^ T淋巴细胞的变化，可对治疗效果起到一定的预测作用[9]。

国内也有中心报道PRCA发病多年后出现淋巴瘤的病例[18]–[20]，提示淋巴瘤和PRCA发病机制存在共性关系。由于PRCA的发病机制涉及免疫异常和治疗药物带来的免疫细胞功能调节，而淋巴瘤作为淋巴系统造血组织的恶性肿瘤，免疫学异常是其重要的发病机制之一，两者在发病机制上存在共同路径，因此，需要在临床诊治中需做好密切随访工作。

PRCA的治疗由于缺乏高质量的循证医学证据，目前并没有统一的推荐方案，治疗决策主要根据患者的病因分类，可选择糖皮质激素、环孢素A、环磷酰胺、西罗莫司等药物治疗以及手术治疗如切除胸腺瘤[13],[21]–[22]。对于继发于淋巴瘤的PRCA，选择针对淋巴瘤的一线化疗能够有效改善病情，必要时加用糖皮质激素能进一步提高疗效。本报道也提示一线采用ECHOP方案化疗对患者的PTCL和PRCA均有效。
